# Robots are judging me: Perceived fairness of algorithmic recruitment tools

**DOI:** 10.3389/fpsyg.2022.940456

**Published:** 2022-07-25

**Authors:** Airlie Hilliard, Nigel Guenole, Franziska Leutner

**Affiliations:** ^1^Institute of Management Studies, Goldsmiths, University of London, London, United Kingdom; ^2^Holistic AI, London, United Kingdom; ^3^HireVue, Inc., London, United Kingdom

**Keywords:** selection, recruitment, fairness, perceptions, psychometrics, algorithm, machine learning

## Abstract

Recent years have seen rapid advancements in selection assessments, shifting away from human and toward algorithmic judgments of candidates. Indeed, algorithmic recruitment tools have been created to screen candidates’ resumes, assess psychometric characteristics through game-based assessments, and judge asynchronous video interviews, among other applications. While research into candidate reactions to these technologies is still in its infancy, early research in this regard has explored user experiences and fairness perceptions. In this article, we review applicants’ perceptions of the procedural fairness of algorithmic recruitment tools based on key findings from seven key studies, sampling over 1,300 participants between them. We focus on the sub-facets of behavioral control, the extent to which individuals feel their behavior can influence an outcome, and social presence, whether there is the perceived opportunity for a social connection and empathy. While perceptions of overall procedural fairness are mixed, we find that fairness perceptions concerning behavioral control and social presence are mostly negative. Participants feel less confident that they are able to influence the outcome of algorithmic assessments compared to human assessments because they are more objective and less susceptible to manipulation. Participants also feel that the human element is lost when these tools are used since there is a lack of perceived empathy and interpersonal warmth. Since this field of research is relatively under-explored, we end by proposing a research agenda, recommending that future studies could examine the role of individual differences, demographics, and neurodiversity in influencing fairness perceptions of algorithmic recruitment.

## Introduction

Recent years have seen rapid advances in the way that pre-employment tests are delivered, with commercial providers offering game- and image-based assessments, on-demand asynchronous video interviews, and tools to screen applicant resumes, all of which use algorithms to make decisions on the suitability of candidates (Albert, 2019; Guenole et al., forthcoming). Recent estimates indicate that over 75% of organizations are currently or are interested in using algorithms in their talent sourcing, with 7% fully automating their approach (Laurano, 2022). Much of the work on algorithmic recruitment tools thus far has focused on performance metrics and bias (e.g., Köchling and Wehner, 2020; Landers et al., 2021; Hilliard et al., 2022a) over fairness, a social construct that is distinct from bias (Society for Industrial and Organizational Psychology, 2018). Perceptions of fairness can concern distributive fairness – fairness in outcomes – or procedural fairness – fairness in the procedure used during an assessment, typically characterized by perceived ability to influence a decision (Gilliland, 1993). Much of the research regarding the perceived fairness of algorithmic recruitment tools investigates procedural fairness since it is a concern shared by both candidates and human resources practitioners (Fritts and Cabrera, 2021; Mirowska and Mesnet, 2021).

In this review, we explore the perceived procedural fairness of algorithmic recruitment tools, where we define an algorithmic recruitment tool as one that combines non-traditional or unstructured data sources with machine learning to predict a job-relevant construct, such as personality (Guenole et al., 2022). Google Scholar was used to source the studies included in this review through searches including key words such as algorithm, algorithmic, hiring, selection, recruitment, fairness perceptions, and perceived fairness. Article abstracts were used to identify the recurring themes of general procedural justice, behavioral control, and social presence. Studies investigating these constructs were therefore included while studies investigating other sub-facets of procedural justice or distributive justice were excluded to limit the scope of the review.

We find that perceptions of general procedural fairness are mixed but when examined at a sub-facet level, perceptions are more negative. Specifically, algorithmic recruitment tools can be perceived as unfair because of their objectivity, which makes it harder for candidates to use impression management and influence decisions, and because there is a lack of human connection. Since research in this field is in a nascent stage, we also suggest a research agenda to better understand perceptions of algorithmic selection assessments, recommending that future research could examine the influence of individual differences and demographics on fairness perceptions, as well as use real-life applicants over hypothetical scenarios.

## Perceptions of algorithmic selection assessments

Although a relatively new research area, some studies have investigated reactions to algorithmic selection assessments. Game-based assessments, for example, offer shorter testing times (Atkins et al., 2014; Leutner et al., 2020) and are perceived as more engaging (Lieberoth, 2015), more satisfying (Georgiou and Nikolaou, 2020), and more immersive (Leutner et al., 2020) than their questionnaire-based equivalents. However, much of the research so far has investigated user experience, rather than fairness perceptions.

While it is of course important to investigate how candidates feel when they interact with these tools, it is also important to investigate the perceptions of these assessments since negative perceptions might discourage candidates from interacting with these tools in the first place, something that is likely to be a major concern for employers given the current labor shortages (Office for National Statistics, 2021). However, perceptions of organizations can be worsened when they use automatic analysis of interviews, and there is a preference for resumes to be screened by humans rather than algorithms (Wright and Atkinson, 2019). To this end, in the following section, we examine the research into the fairness perceptions of algorithmic recruitment tools. We start by examining perceptions of procedural fairness as a whole before focusing on the sub-facets of behavioral control and social presence. We provide an overview of the key themes and findings of the seven key studies we discuss in Table 1.

**TABLE 1:** A summary of the key findings of the seven key studies discussed.

Citation	Tool	Participants	Key findings
Georgiou and Nikolaou, 2020	Game-based SJT	73 employees of an IT company (+88 control); 131 students/alumni of a South-European university	Higher levels of process satisfaction and organizational attractiveness for the game-based SJT compared to the traditional form Higher levels of perceived fairness through process satisfaction
Suen et al., 2019	Video interviews	180 members of a non-profit HR organization in China	No difference in the fairness perceptions of synchronous and asynchronous video interviews No difference in the fairness perceptions of human versus algorithmic rater
Köchling et al., 2022	AI-support in screening and interviews	160 German employees	Decreased perceived opportunity to perform when AI-support used for telephone or video interviews No effect of AI-support in earlier screening stages on perceived opportunity to perform
Langer et al., 2019	Video interviews	123 German participants	Automated interviews in the selection context is associated with less perceived behavioral control and lower levels of acceptance through lower social presence compared to automation in low-stakes context or synchronous video interviews
Mirowska and Mesnet, 2021	Artificial intelligence evaluations	33 French professionals	Interviews found that participants accepted algorithms to be more objective than humans but nevertheless preferred human judgments, despite them being prone to bias
Kaibel et al., 2019	Screening tool; online assessment, video interview	165 German employees; 255 American MTurk workers	Across all of the tools, perceived opportunity to perform and social presence was lower for algorithmic judgments compared to human judgments
Lee, 2018	Screening tool	228 American MTurk workers	Human decisions judged as fairer than algorithmic as algorithms lack human intuition and cannot make exceptions

## Algorithmic recruitment and procedural fairness

When examined at an overall level, perceptions of procedural fairness of algorithmic recruitment tools are typically positive, although this can vary by assessment tool. For example, Georgiou and Nikolaou (2020) report that game-based situational judgment tests, which are scored using an algorithm, are viewed as fairer than traditional situational judgment formats. In contrast, Suen et al. (2019) report that although there is a preference for synchronous video interviews compared to asynchronous, fairness perceptions do not vary when asynchronous video interviews are judged by humans compared to an algorithm. More recent research reveals further contrasts, with algorithmic tools being seen as less fair when used in later stages of the recruitment funnel and equally as fair as human ratings when used in earlier stages, such as resume screening (Köchling et al., 2022).

While perceptions of procedural fairness can be examined using a broad definition, as was the approach of Suen et al. (2019) and Georgiou and Nikolaou (2020), they can also be examined at a more granular level. Indeed, procedural fairness can be examined in relation to specific variables including social presence, interpersonal treatment, perceived behavioral control, and consistency (Langer et al., 2019). Examining procedural fairness at this level can provide greater insight into the factors driving fairness perceptions, and how applicants interact with the tools. In the following sub-sections, we examine procedural fairness perceptions in relation to the sub-facets of social presence, referring to the perceived ability to form social connections and experience empathy during the application experience, and perceived behavioral control, referring to the extent to which an individual believes they can control or influence an outcome with their behavior (Langer et al., 2019) to reflect the elements of procedural fairness most frequently investigated in the literature.

### Algorithmic recruitment and behavioral control

In contrast to the mixed findings of overall procedural fairness, when procedural fairness is examined at a more granular level, fairness perceptions of algorithmic recruitment tools are more consistent. Several studies report that across different algorithmic recruitment tools, there is less of an opportunity to perform. In other words, participants perceive less behavioral control. Indeed, despite being seen as more objective, algorithmic asynchronous video interviews are seen as having less opportunity to form in comparison to human-rated asynchronous video interviews (Kaibel et al., 2019). Likewise, algorithmic resume screening tools are perceived to be less able to judge human character compared to when humans screen resumes (Lee, 2018), despite humans spending less than 10 seconds reading each resume (Ladders, 2018), and being biased against non-white applicants (Bertrand and Mullainathan, 2004). Specifically, applicants perceive it to be unfair that algorithms cannot make exceptions whereas human raters can (Lee, 2018). This indicates that applicants perceive that there is less opportunity for them to perform with algorithmic judgments because of their objectivity, meaning that they are less able to manipulate the algorithm than human raters. Therefore, efforts to make recruitment funnels more objective and standardized, and therefore fairer in terms of a lack of bias, has resulted in them being perceived as more unfair.

A possible explanation for applicants perceiving less opportunity to perform might be influenced by a lack of knowledge about what the algorithm uses to make judgments, particularly because algorithms are typically considered black-box or glass-box systems (Cheng and Hackett, 2021), meaning the internals of the model are uninterpretable or unknown (Guidotti et al., 2018). However, providing some explanation of how the algorithms make decisions to candidates does not always improve perceptions (Langer et al., 2018), and can even worsen perceptions of fairness and organizational attractiveness by invoking concerns about privacy due to the data being used to compute judgments (Langer et al., 2021). This suggests that it is not the lack of knowledge about algorithmic decision-making that drives lower perceptions of opportunity to perform with algorithmic tools, but the fact that candidates are more confident in influencing human decision-making since the factors that humans consider when making judgments are more intuitive and superficial compared to datapoints used by algorithms. Therefore, the more subjective nature of human judgments is preferred because participants believe that they are more able to influence the decision of their rater through impression management, which is not uncommon for candidates to use during their application (e.g., Weiss and Feldman, 2006). Since human biases are notoriously difficult to address (Atewologun et al., 2018) and a properly trained algorithm that has been tested for bias is likely to result in fewer sub-group differences (Lepri et al., 2018), impression management is less likely to have an effect on algorithmic decision tools.

### Algorithmic recruitment and social presence

Perceptions of social presence are concerned with the extent to which applicants perceive there to be an interpersonal connection facilitated by empathy and warmth during an interaction (Langer et al., 2019). Investigations of this aspect of procedural fairness have found that algorithmically analyzed video interviews and tests of performance are rated as less personable, or lower in social presence, than manual ratings, despite applicants not interacting with a human in either condition (Kaibel et al., 2019). This suggests that algorithmic judgments are perceived as being unfair as they are less able to reflect human values and replicate interpersonal exchanges as an algorithm cannot empathize with candidates like humans can. Indeed, despite acknowledging that algorithmic recruitment tools are more objective than human ratings, and that human ratings can be biased, participants still report perceiving algorithmic recruitment tools are less fair due to the lack of human connection and interaction (Mirowska and Mesnet, 2021). This could explain why algorithmic tools used earlier in the funnel, where there is typically less human interaction, are seen as equally fair to human ratings, while algorithmic tools used later in the funnel, such as during the interview stage, are viewed as less fair than human ratings (Köchling et al., 2022) since there are differences in the level of human connection expected.

Concerns about lack of opportunity to perform are also echoed by human resource (HR) practitioners who believe that algorithms have artificial instead of human values. This is because the relationship between humans is very different to the relationship between humans and computers as the latter can become gamified and lack sincerity (Fritts and Cabrera, 2021). Removal of or limiting the human element in the recruitment process also has implications for the connections that recruiters can form with candidates (Li et al., 2021), particularly if much of the process is automated and recruiters are only involved in making the final decision based on algorithmic recommendations.

## A research agenda

While the findings discussed above are an important step in the right direct toward understanding perceptions of algorithmic recruitment tools, this area of research is still in its infancy and there are a number of limitations with these studies and additional factors that might influence perceptions to still be explored. Indeed, a major limitation with these studies is that they use hypothetical scenarios instead of real-life applicants. Although Langer et al. (2019) made some progress toward this by framing the video interviews they used in their study as high-stakes (selection scenario) or low-stakes (training scenario), there is still a lack of research using real-life applicants. We therefore propose that future studies should examine perceptions among real-life applicants, perhaps by partnering with a commercial provider assisting an employer implementing automated approaches for the first time. This would allow between-group comparisons to take place, where perceptions of applicants completing the traditional process and the newly introduced automated process could be compared.

Existing research also does little to investigate whether perceptions of automated tools are influenced by demographic or neurological differences. To this end, we propose that future studies could investigate whether age influences perceptions of fairness since technology self-efficacy decreases with age (Hauk et al., 2018; Ellison et al., 2020), meaning that older applicants may perceive particularly low levels of fairness. Future research could also examine whether fairness perceptions of algorithmic recruitment tools differ by racial group. This is because both human and algorithmic judgments may negatively impact non-white candidates; humans are notorious for being biased when screening resumes (Bertrand and Mullainathan, 2004) and facial recognition (Buolamwini, 2018) and voice recognition technologies (Bajorek, 2019), which are used during analysis of video interviews, are less accurate for darker-skinned individuals. Future studies could, therefore, investigate whether human or algorithmic judgments are more favored among non-white applicants, given that there is the potential for discrimination with both avenues. Additionally, perceptions among neurodivergent populations, such autistic applicants, could also be examined, particularly since much of the research investigating neurodivergence and employment relates to support and coaching during job search, rather than experiences with pre-employment tests (e.g., Baldwin et al., 2014; Fontechia et al., 2019). Research that has examined autism in relation to pre-employment tests resulted in mixed findings, with one battery of game-based cognitive ability assessments resulting in no significant difference in the performance of autistic and neurotypical respondents and the second resulting in neurotypical respondents performing better than autistic (Willis et al., 2021). To extend these findings, further research could investigate the fairness perceptions of autistic candidates instead of performance, exploring how they may or may not benefit from the social element being removed or limited. Investigation of these suggested factors would provide important insights into whether certain groups might be disproportionately discouraged from applying to positions where candidates will be evaluated by algorithmic tools.

Individual differences in personality is another area that is underexplored in the currently available research that might help to explain some of the mechanisms underlying algorithmic skepticism in recruitment. Specifically, the influence of emotional intelligence, Machiavellianism, and the Big Five traits on fairness perceptions might provide some particularly useful insights. This is because emotional intelligence is characterized by being able to control the emotions of others as well as one’s own emotions (Salovey and Mayer, 1990) and is related to the selective presentation of information and behavior (Kilduff et al., 2010), meaning that individuals high in emotional intelligence may feel that there is less opportunity to perform with an algorithmic tool since they cannot manipulate these as easily as humans. Likewise, Machiavellianism is associated with selective self-presentation and manipulation of others to achieve goals (Kessler et al., 2010) and algorithmic recruitment tools are perceived as less sensitive to social cues and less likely to make exceptions (Lee, 2018), which may result in them being disfavored by those high in Machiavellianism. These influences are yet to be investigated by the literature. Although these factors are yet to be investigated in the literature, there have been some efforts toward investigating the influence of the Big Five, with Georgiou and Nikolaou (2020) finding no influence of openness to experience on fairness perceptions. However, there is a lack of research to corroborate this finding or investigating the remaining traits. We, therefore, recommend that future research could investigate the influence of the Big Five traits on perceptions, at both an overall and a facet level.

Finally, future research could examine the influence of explanation on perceptions since although explanations have little effect on organizational attractiveness (Langer et al., 2018) or can in fact worsen perceptions of fairness due to privacy concerns, providing a justification for why the tool is being used can mitigate this (Langer et al., 2021). Further, since negative perceptions of algorithmic recruitment tools can occur when candidates are given explanations about algorithmic tools due to concerns about privacy (Langer et al., 2021), further research could investigate how to overcome these concerns, particularly since concerns about privacy can influence whether or not practitioners are willing to adopt these tools (Guenole et al., forthcoming). The introduction of the Artificial Intelligence Video Interview Act (820 ILCS 42, 2020) and the passing of mandatory bias audits in New York City (Int 1894-2020, 2021) – which require that the features being used by algorithms be disclosed to candidates, facilitating greater informed consent for the use of the tool (Hilliard et al., 2022b) – could provide an opportunity to further examine how the framing of explanations influences fairness perceptions, which might inform practical recommendations regarding explaining tools to candidates.

## Conclusion

While applicant reactions to algorithmic selection assessments, particularly game-based assessments, are generally positive (Atkins et al., 2014; Lieberoth, 2015; Georgiou and Nikolaou, 2020; Leutner et al., 2020), the same cannot be said for fairness perceptions. At a general level, fairness perceptions are mixed and are not consistent between assessment tools (Suen et al., 2019; Georgiou and Nikolaou, 2020; Köchling et al., 2022) but when looking at specific aspects of procedural justice, algorithmic tools are perceived as less fair than human ratings. Indeed, candidates perceive that there is less opportunity for behavioral control when assessments are automated compared to when they are judged by humans, meaning that they feel they are given less chance to perform and manipulate the raters to influence them toward a positive judgment (Lee, 2018; Kaibel et al., 2019). Candidates also perceive that there is less social presence when recruitment processes are automated (Kaibel et al., 2019; Mirowska and Mesnet, 2021), a view that is also shared by HR professionals (Fritts and Cabrera, 2021; Li et al., 2021). However, research into fairness perceptions of algorithmic recruitment tools is still in its infancy and there is scope for further investigations, which could help to inform how fairness perceptions might be improved, particularly for groups that are already underrepresented in organizations and application processes. As such, we suggest a research agenda for exploring this further, recommending that future studies should use real-life applicants and could investigate the role of individual differences in personality and emotional intelligence as well as age and race in influencing fairness perceptions, and how explanations of the algorithms should be framed. Given the current labor market shortages (Office for National Statistics, 2021) and the importance of perceptions of an organization’s recruitment process in influencing whether candidates accept a job offer (Hausknecht et al., 2004), improving fairness perceptions of algorithmic recruitment tools has significant implications for both candidates and organizations and is, therefore, an important area for further investigation.

## Author contributions

AH was responsible for writing the first draft of the manuscript. FL and NG were responsible for writing and editing the manuscript. FL supervised the manuscript. All authors were responsible for conceptualization.

## Conflict of interest

AH and FL were employed by Holistic AI and HireVue, respectively. The remaining author declares that the research was conducted in the absence of any commercial or financial relationships that could be construed as a potential conflict of interest.

## Publisher’s note

All claims expressed in this article are solely those of the authors and do not necessarily represent those of their affiliated organizations, or those of the publisher, the editors and the reviewers. Any product that may be evaluated in this article, or claim that may be made by its manufacturer, is not guaranteed or endorsed by the publisher.
